# Excess weight and thinness over two decades (1996–2015) and spatial distribution in children from Jujuy, Argentina

**DOI:** 10.1186/s12889-021-10239-4

**Published:** 2021-01-22

**Authors:** María José Bustamante, Emma Laura Alfaro, José Edgardo Dipierri, María Dolores Román

**Affiliations:** 1 Instituto de Ecorregiones Andinas, Consejo Nacional de Investigaciones Científicas y Técnicas – Universidad Nacional de Jujuy, Avenida Bolivia 1239, CP 4600 San Salvador de Jujuy, Argentina; 2 Instituto de Biología de la Altura, Universidad Nacional de Jujuy, 1661 Bolivia Avenue, CP 4600 San Salvador de Jujuy, Argentina; 3 Centro de Investigaciones en Nutrición Humana, Escuela de Nutrición, Facultad de Ciencias Médicas, Universidad Nacional de Córdoba, Boulevard de la Reforma s/n, CP 5000 Córdoba, Argentina

**Keywords:** Obesity, Thinness, Prevalence, Trends, Spatial distribution, Children, Jujuy

## Abstract

**Background:**

The increase of excess weight around the world is progressive and sustained in children. This is the most prevalent form of malnutrition in this population and they represent the major public health problem in developed and developing countries. The aim of this study was to analyze the magnitude of change in thinness and excess weight prevalence in 4–7 years-old schoolchildren from Jujuy (Argentina), between 1996 and 2015 and to examine the association according to sex and school location.

**Methods:**

Cross-sectional study. Data was obtained from databases of School Health programs and it is representative of the city school population. For the analysis, 31,014 schoolchildren between 4 and 7 years old were evaluated, 20,224 from the first period (1996–2001) and 10,790 from the second (2010–2015). The city was partitioned in three different areas determined by the rivers that cross it. Nutritional status was determined by BMI for age with the criteria suggested by the International Obesity Task Force. The percentage of malnutrition change between periods was calculated and a binomial regression model was adjusted.

**Results:**

Between periods, a significant (*p*-value< 0.0001) increase in the prevalence of overweight from 15.1% (CI 14.6–15.6%) to 18.1% (CI 17.4–18.8%) and obesity from 5% (CI 4.7–5.3) to 10.7% (CI 10.1–11.3%), and a decrease of thinness prevalence from 6.3% (CI 6.0–6.7%) to 4.7% (CI 4.3–5.1%) were observed. The percentage of change in the prevalence of obesity was very high in all areas and in both sexes (103.5% girls; 125.6% in boys), being higher in the south for girls (122.4%) and in the north for boys (158.8%). Besides, being a boy was inversely associated with the presence of excess weight and, as the age increases, the presence of obesity does it too. By analyzing the effect of the school location, the south and north zones had an inverse association with the presence of obesity. The period has a direct association with the presence of excess weight.

**Conclusion:**

The study contributes with valuable information on the magnitude of the increase in obesity in schoolchildren and suggests a possible correlation with sex and spatial distribution in the capital city of Jujuy.

## Background

Globally, the prevalence of childhood obesity, overweight and thinness varies over time and according to the region and country [1, 2]. Currently, more than 20% (approximately 42.5 million) of Latin American children aged between 0 to 19 years old are overweight or obese [3]. In Latin America, strikingly high prevalence of excess weight is observed in children under 5 years of age and such prevalence is still rising [3].

In Argentina, according to the National Survey of Nutrition and Health 2004/5 (ENNyS, due to its acronym in Spanish), in children population under 5 years old, the prevalence of excess weight was 31.5% (10,4% corresponding to obesity). No differences were observed between sexes [4]. More recently, the ENNyS 2018/9 showed a prevalence of 20.7% of overweight and 20.4% of obesity in 5 to 17 years old population, being obesity higher in males [5].

In consonance with all the national surveys, the increase of overweight and obesity around the world is progressive and sustained in children and adolescents. These are the most prevalent forms of malnutrition in these populations in which deficit malnutrition and excess malnutrition coexist [5, 6].

This situation is related to rapid urbanization combined with diets that rely on energy-dense, nutrient-poor foods and sedentary behaviors that have become the norm among children [3]. In addition, malnutrition in general tends to be strongly associated with gender and socioeconomic status but the direction of these associations varies according to the levels of economic development of the places [7, 8]. So, poverty, specifically chronic poverty, is identified as one of the root causes of deficit malnutrition and food insecurity, which is also one of the most noticeable symptoms of economic and social inequality [9].

Besides, the nutritional situation in South America is the consequence of three concurrent processes: the nutritional transition, the economic crises of recent years, and unsolved chronic problems [10]. In this context, Argentina presents an advanced state of demographic, epidemiological and nutritional transition and is one of the countries with the highest excess weight [10].

These are not only problems that affect growth and development of children and adolescents. They can also cause other problems or complications, such as psychological disorders and cognitive dysfunction, influencing the timing of puberty, and they may be accompanied by metabolic syndrome [11]. This is an alarming situation due to a number of diseases acquired during this period may carry over to adulthood or are risk factors for adult diseases, like prediabetes and cardiovascular diseases. Besides, they are strongly associated with mortality in middle age [11–15].

To generate effective intervention strategies aimed to prevent the obesity epidemic, it is essential to study factors related to the nutritional status of the populations. Winkelstein has pointed out that “ecological factors may be the most important determinants of the health and disease status of a population” [16, 17]. The neighborhoods in which people live may influence health, operating through mechanisms such as: the availability and accessibility of health services; infrastructure deprivation, the prevailing attitudes towards health and related behaviors, stress and a lack of social support [17].

The distribution of these characteristics in the urban space is heterogeneous and this is clearly shown in the capital city of Jujuy (San Salvador de Jujuy), where a clear decline in the socioeconomic conditions from the center to the periphery is observed [18]. Fournier (2002) describes different levels of socioeconomic segregation in the urban space of Jujuy. Level 1: It is characterized by the differentiation of social classes depending on the distance to the center (in the immediate vicinity of the functional center of the city are the traditional houses of upper-class families). Level 2: It is shown in the form of a decline of the social structure of the neighborhoods in the north-south direction: towards the north, in the highest places and on the other side of the Rio Grande, the big single-family houses dominate; the sectors located to the south of the center are characterized by popular districts with a much higher density of inhabitants. Level 3: It manifests itself in the differentiation of dwellings in terms of their location with respect to height (in the city located in a fluvial valley, the highest areas are populated by the upper classes while the socially marginalized settle at the edges of the rivers) [18, 19].

To monitor trends of thinness and excess weight, it is fundamental to analyze the magnitude of changes over time, in order to explore possible risks factors and to identify subgroups of more affected populations. In addition, the analysis of the relationship between prevalence and spatial distribution is especially important as it contributes to the understanding of malnutrition not as an isolated, but complex phenomenon, in which various environmental and contextual factors intervene. Thus, these considerations must be taken into account to generate intervention strategies in the field of public health that are appropriate to the characteristics of the target population.

The aim of this study was to analyze the magnitude of the change in the thinness and excess weight prevalence in 4–7 year-old schoolchildren from San Salvador de Jujuy (Jujuy Province, Argentina) between 1996 and 2000 and 2010–2015 and to examine the association according to the sex and the school location.

## Methods

### Study settings

**Fig. 1:**
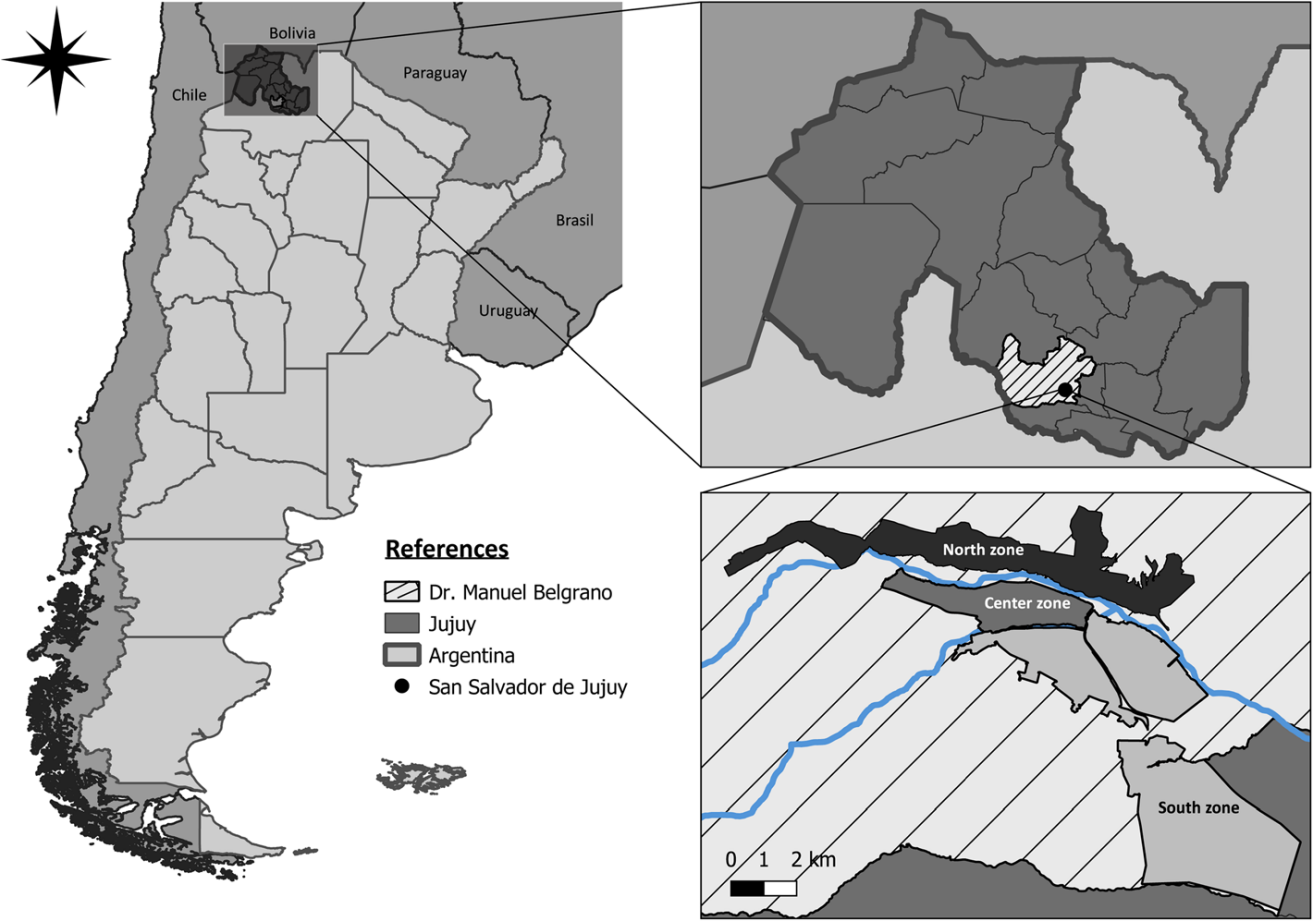
Geographic location of San Salvador de Jujuy in Jujuy province and in Argentina. Source: Own elaboration using QGIS 3.6

This study was conducted in San Salvador de Jujuy, capital city of Jujuy Province (Fig. 1), located in Doctor Manuel Belgrano Department in the Valley Region of the Province (Northwestern Argentina), characterized by mesothermal climate and an average altitude of 1100 masl. According to the 2010 census, San Salvador de Jujuy concentrates 38.3% of the province population. Besides, of the total 4–7 year-old urban departmental population, 85.7% attends school regularly, 1.8% is illiterate, and 12.7% lives in home with unsatisfied basic needs (UBN) [20, 21].

The total population for the Doctor Manuel Belgrano Department in 2001 was 238,012, of which 21,603 were from the 4–7-year-old population. In the 2010 census the population of the department was 265,249, of which 17,987 had 4–7 years old [20, 21].

### Data and measurements

The National School Health Program is developed as an “Integrated Care Policy for children and adolescents” between the National Ministries of Health, Education and Sports. This program was implemented in the province of Jujuy in 2010. However, prior to that, in the city of San Salvador de Jujuy, scholar children were already being evaluated in the School Health Department of the Ministry of Social Welfare of the province.

The aim of these programs is to assess children nutritional status and health. Anthropometric nutritional evaluation by these programs for all schoolchildren who enter the primary school system in the city of San Salvador de Jujuy is mandatory. Therefore, these data represent the majority of individuals who enter this educational level. So, the study population was all the schoolchildren between 4 and 7 years old, who attend public and private schools in San Salvador de Jujuy, evaluated in two periods (1996–2000 and 2010–2015).

**Fig. 2:**
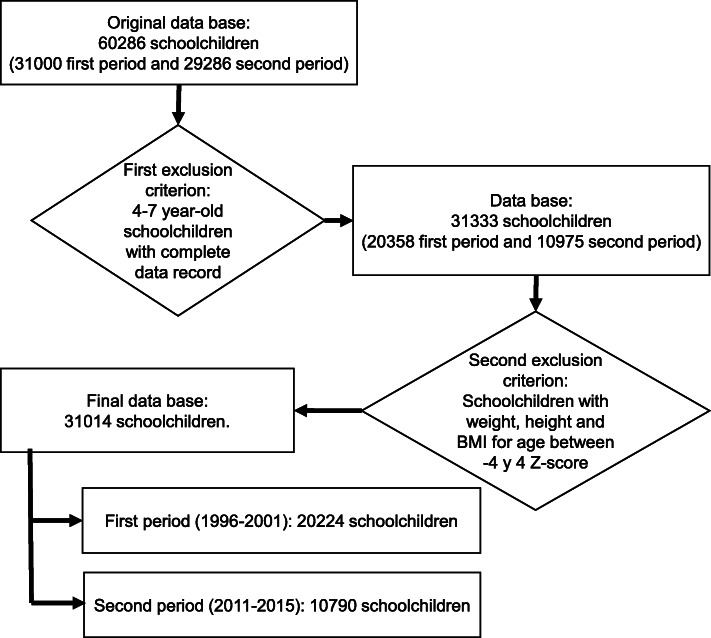
Data-Flow Diagram of the School Health Program data bases from San Salvador de Jujuy

The information gathered and analyzed in this study (weight -kg-, height -cm- and school location) comes from the databases of these programs. These anthropometric measurements were taken by health personnel trained for this task. Schoolchildren with missing data on age, sex, weight and height, as well as individuals with extreme weight, height or body mass index (BMI) for age (± 4 Z-score) related to inadequate data recording were excluded (Fig. 2).

In order to examine possible associations between the nutritional status of schoolchildren and the place where the schools are located, these were grouped into three zones. The capital city schools were categorized according to their location in relation to the two main rivers that cross the city: a) North: to the north of Río Grande; b) Center: between the two rivers (Río Grande and Río Xibi-Xibi); c) South: to the south of Río Xibi-Xibi. This grouping reflects quite well the level 1 segregation in the Jujuy urban space described by Fournier [19].

### Analyses

Nutritional status was determined in accordance with the criteria and cut-off points suggested, for BMI for age, by the International Obesity Task Force to establish normalweight, thinness, overweight and obesity phenotypes, according to sex and age [22].

Prevalence and 95% confidence intervals (95%CI) of thinness, overweight and obesity by sex and school location were calculated for each period and the comparisons between proportions were analyzed with Chi Square.

The differences between periods in the prevalence of the different nutritional status (Prevalence 2nd period - Prevalence 1st period) and the percentage of change (Difference between periods * 100 / Prevalence 1st period) were calculated. Furthermore, a binomial regression model was adjusted for each malnutrition outcome (thinness, overweight and obesity), including sex, age, school location and period as covariates.

The statistical program Stata v14 was used for data description and analysis.

## Results

After the exclusion criteria were applied, the sample size was 31,014, 20,224 from the first period (1996–2001) and 10,790 from the second (2010–2015). The mean of age was higher in the second period (6.6 ± 0.4 years old) in relation to the first one (5.5 ± 0.4 years old). In both periods, the distribution between sexes was similar. In terms of malnutrition, a high prevalence of excess weight was observed in both periods. In relation to extreme categories of nutritional status, higher thinness was observed in the first period and obesity in the second (Table 1).

**Table 1:** Distribution of children by sex, school location and nutritional status, according to period

VARIABLES		1996–2001		2010–2015		p-value
		% (95%CI)	n	% (95%CI)	n	
**SEX**	**Boys**	49.60 (48.9–50.3)	10,040	48.40 (47.5–49.4)	5227	0,0441
	**Girls**	50.40 (49.7–51.0)	10,184	51.60 (50.6–52.5)	5563	0.0441
**ZONE**	**Center**	29.70 (29.1–30.4)	6015	43.30 (42.3–44.2)	4668	< 0.0001
	**North**	8.50 (8.1–8.8)	1712	15.10 (14.5–15.8)	1634	< 0.0001
	**South**	61.80 (61.1–62.5)	12,497	41.60 (40.7–42.5)	4488	< 0.0001
**NUTRITIONAL STATUS**	**Thinness**	6.30 (6.0–6.7)	1278	4.70 (4.3–5.1)	508	< 0.0001
	**Normalweight**	73.60 (73.0–74.2)	14,880	66.50 (65.6–67.4)	7173	< 0.0001
	**Overweight**	15.10 (14.6–15.6)	3061	18.10 (17.4–18.8)	1955	< 0.0001
	**Obesity**	5.00 (4.7–5.3)	1005	10.70 (10.1–11.3)	1154	< 0.0001

Table 2 shows the prevalence and 95%CI of thinness, overweight and obesity according to the school location, period and sex. As it can be seen, in relation to differences between zones, in both periods, girls from the south and north had a significantly lower prevalence of obesity than girls from the center zone (*p*-value < 0.05). In boys, no differences in prevalence between zones were observed in any period.

**Table 2:** Prevalence of nutritional status categories by sex and period according to school location

NUTRITIONAL STATUS		SCHOOL LOCATION % (95%CI)			TOTAL % (95%CI)
		CENTER	NORTH	SOUTH	
**1996–2000** **GIRLS**	**Thinness**	5.4 (4.6–6.2)	5.3 (3.9–7.1)	6.2 (5.6–6.8) ^#^	5.9 (5.4–6.3) ^#^
	**Normal weight**	70.7 (69.1–72.3) ^#^	74.8 (71.7–77.7)*	73.1 (72.0–74.2)* ^#^	72.5 (71.6–73.4) ^#^
	**Overweight**	16.6 (15.3–17.9)	14.8 (12.5–17.4)	15.8 (14.9–16.7) ^#^	15.9 (15.2–16.7) ^#^
	**Obesity**	7.3 (6.4–8.2) ^#^	5.0 (3.6–6.7)*	4.9 (4.4–5.5)*	5.7 (5.2–6.1) ^#^
		*n* = 3156	*n* = 822	*n* = 6206	*n* = 10,184
**1996–2000** **BOYS**	**Thinness**	6.5 (5.6–7.5)	5.6 (4.2–7.3)	7.1 (6.4–7.7)	6.8 (6.3–7.3)
	**Normal weight**	73.6 (72–75.2)	76.5 (73.6–79.2)	74.8 (73.7–75.9)	74.6 (73.8–75.5)
	**Overweight**	15.2 (13.9–16.6)	14.5 (12.2–16.9)	13.9 (13–14.7)	14.3 (13.6–15)
	**Obesity**	4.6 (3.9–5.4)	3.4 (2.3–4.9)	4.2 (3.7–4.8)	4.3 (3.9–4.7)
		*n* = 2859	*n* = 898	*n* = 6283	n = 10,040
**2010–2015** **GIRLS**	**Thinness**	5 (4.1–5.9)	6.2 (4.7–8.1)	3.8 (3.1–4.7) ^&^	4.7 (4.1–5.3) ^&^
	**Normal weight**	64.1 (62.2–66)^&^	65.2 (61.8–68.4) ^&^	66.6 (64.7–68.6) ^&^	65.3 (64.1–66.6) ^& #^
	**Overweight**	18.1 (16.6–19.7)	18.4 (15.8–21.2) ^&^	18.6 (17–20.2) ^&^	18.4 (17.4–19.4) ^&^
	**Obesity**	12.7 (11.4–14.1) ^& #^	10.2 (8.2–12.4)* ^&^	10.9 (9.6–12.2)* ^&^	11.6 (10.8–12.5) ^& #^
		*n* = 2415	*n* = 836	*n* = 2312	*n* = 5563
**2010–2015** **BOYS**	**Thinness**	5.3 (4.4–6.3) ^&^	4.5 (3.2–6.2)	4.2 (3.4–5.2) ^&^	4.7 (4.2–5.3) ^&^
	**Normal weight**	66.3 (64.3–68.3) ^&^	68.7 (65.4–71.9) ^&^	68.7 (66.7–70.7) ^&^	67.7 (66.4–68.9) ^&^
	**Overweight**	18.5 (17–20.2) ^&^	18 (15.4–20.9) ^&^	17.1 (15.5–18.7) ^&^	17.8 (16.8–18.9) ^&^
	**Obesity**	9.8 (8.6–11.1) ^&^	8.8 (6.9–10.9) ^&^	10.0 (8.7–11.3) ^&^	9.7 (8.9–10.6) ^&^
		*n* = 2253	*n* = 799	*n* = 2175	*n* = 5227

Significance *p* < 0.05: *Vs Center – ^#^Vs Boys – ^&^Vs 1996–2000

**Fig. 3:**
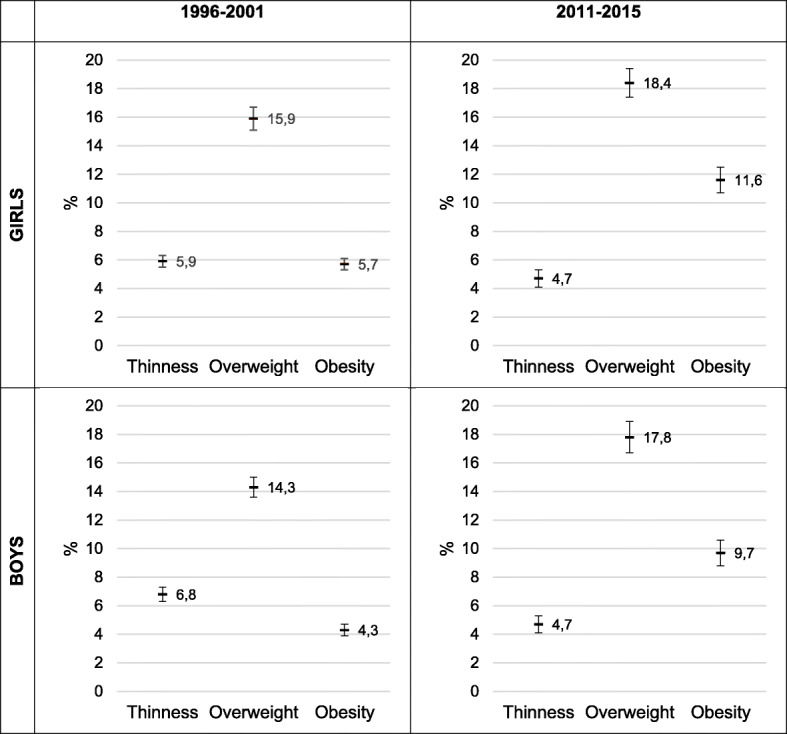
Prevalence and IC of thinness, overweight and obesity for period and sex

Differences between sexes in each period and zone only were observed in the prevalence of obesity in the center zone, being higher in girls. By comparing the total prevalence between periods, significant differences were observed, being higher the prevalence of overweight and obesity in the second period in girls and boys in almost all zones but with a variable magnitude. In addition, to improve the general interpretation of these results for period and sex, in Fig. 3 it can be seen graphically the prevalence and CIs for thinness, overweight and obesity.

As it can be seen in Table 3, the magnitude of the increase in the prevalence of obesity is very high in all areas and in both sexes (103.5% in girls and 125.6% in boys). This increase was higher in the south (122.4%) for girls and in the north (158.8%) for boys. In relation to overweight, the increase is not as high as obesity, being 15.7% in girls and 24.5% in boys, higher in the south in both cases (24.3 and 24.1%, respectively). On the other hand, the magnitude of decrease in the prevalence of thinness was 20.3% in girls and 30.9% in boys. The most marked decrease occurred in the south for both, girls (38.7%) and boys (40.8%).

**Table 3:** Magnitude (%) of rate change of obesity, overweight, normal weight and thinness between periods

NUTRITIONAL STATUS		SCHOOL LOCATION			TOTAL
		CENTER	NORTH	SOUTH	
**GIRLS**	**Thinness**	−7.4	17.0	−38.7	−20.3
	**Normal weight**	−9.3	−12.8	−8.9	− 9.9
	**Overweight**	9.0	24.3	17.7	15.7
	**Obesity**	74.0	104	122.4	103.5
**BOYS**	**Thinness**	−18.5	−19.6	−40.8	−30.9
	**Normal weight**	−9.9	−10.2	−8.2	−9.2
	**Overweight**	21.7	24.1	23.0	24.5
	**Obesity**	113.0	158.8	138.1	125.6
**TOTAL**	**Thinness**	−13.5	−3.1	−38.8	−25.5
	**Normal weight**	−9.6	−11.5	−8.6	−9.6
	**Overweight**	14.9	24.4	20.6	19.7
	**Obesity**	89.2	128.7	127.7	115.3

*The magnitude of rate change was expressed like the percentage of change in the prevalence in relation to the first period

Table 4 shows the associations between each malnutrition outcome with the select covariates. Being a boy has been associated to a higher occurrence of thinness (OR = 1.12; 95%CI: 1.02–1.23) and a lower occurrence of overweight (OR = 0.91; 95%CI: 0.86–0.97) and obesity (OR = 0.78; 95%CI: 0.72–0.86). In turn, as the age increases, the occurrence of thinness decreases (OR = 0.85; 95%CI: 0.75–0.95) and obesity increases (OR = 1.17; 95%CI: 1.05–1.30). The school location was not associated with the occurrence of thinness. Although, it was with the occurrence of overweight, being lower in the south (OR = 0.94; 95%CI: 0.88–0.99) and with the occurrence of obesity, being lower in the north (OR = 0.76; 95%CI: 0.66–0.89) and south zones (OR = 0.84; 95%CI: 0.76–0.93) in relation to the center zone. Besides, the second period (2010–2015) was associated to a higher occurrence of overweight (OR = 1.21; 95%CI: 1.09–1.34) and obesity (OR = 1.87; 95%CI: 1.61–2.18) in relation to the first period.

**Table 4:** Associations of thinness, overweight and obesity with sex, age, school location and period

	OR (95% CI)		
	THINNESS	OVERWEIGHT^**#**^	OBESITY
**SEX**			
Girls (reference)	–	–	–
Boys	1.12* (1.02–1.23)	0.91* (0.87–0.96)	0.78* (0.72–0.86)
**AGE (years)**	0.85* (0.75–0.95)	1.02* (0.96–1.09)	1.17* (1.05–1.30)
**ZONE**			
Center (reference)	–	–	–
North	0.98 (0.82–1.16)	0.94 (0.86–1.02)	0.76* (0.66–0.89)
South	1.00 (0.90–1.11)	0.94* (0.88–0.99)	0.84* (0.76–0.93)
**PERIOD**			
1996–2001 (reference)	–	–	–
2010–2015	0.89 (0.75–1.06)	1.23* (1.12–1.34)	1.87* (1.61–2.18)

*OR* Odds Ratio; *CI* confidence intervals

*p-value < 0.05

^#^vs children without excess weight (overweight+obesity)

## Discussion

The magnitude of the increase in the obesity prevalence from 1996 to 2000 to 2010–2015 periods was studied and alarming results was observed. This situation arises not only for the magnitude of this increase, regardless of sex around 20% for overweight and 115% for obesity, but also because occurs in a relatively short time (a decade between periods) in a children population. In addition to this, different systematic reviews suggest that size and growth during infancy are related to risk of obesity in adults, so infants in the highest end of the distribution for weight or BMI and those who grow rapidly are at increased risk of obesity in childhood and adulthood [23–25].

In Latin America, there is a double burden of malnutrition among children that importantly differ between and within countries. Besides, in this study it can be observed that when the age increases, the prevalence of excess weight increases. This phenomenon is observed in Latin American children too [3].

Rapid urbanization combined with greater penetration of the retail food has promoted diets that rely on energy-dense, nutrient-poor foods. At the same time, sedentary behaviors have become the norm among children [3]. In addition to this, Jujuy is located in the region of Argentina where more than 40% of its population is poor and more than 15% are below the extreme poverty line. The region in general has the lowest levels of economic activity, the highest percentage of poverty and indigence, the highest rates of maternal and child mortality, and the worst sanitary conditions in the country [10].

Stabilization of trends is observed in some developed countries, with reversal of trends seen in a select few [14]. The reasons for this apparent plateau observed in the prevalence of obesity among children from an ever-growing number of countries remain unclear. However, it could be hypothesized that obesity rates have reached a country specific ceiling [26]. Another potential explanation is that the cut-off points used for defining BMI categories need re-evaluation under the light of current metabolic and anthropometric data [26]. It is not the case of our country, where the prevalence of overweight and obesity are still rising, as can be seen in this study. However, it should be noted that there are still global trends in the prevalence of obesity that do not respond to ethnic and cultural issues since they are observed in countries that are very different from each other.

At local level, in San Salvador de Jujuy, a study from 1995 to 2000 [27] showed an increase in the prevalence of overweight and obesity in 4–10 year-old children from 13.4 to 17.5%, and 3.7 to 6.7%, respectively, which was similar to what was found in its first period. After that, a multicentric study was conducted in 2007,which revealed a prevalence of 9% of overweight and 4.25% of obesity in children between 6 and 7 years old from Jujuy [6]. Such values are far below to the ones found in this study. On the other hand, data from a program called SUMAR, that provides health care throughout the national territory (Argentina) to pregnant women, children and adolescents up to 19 years old, women and men up to 64 years old, without social coverage, showed similar prevalence of excess weight in Jujuy in 2016 (around 27.7–38.1%) in children between 2 and 9 years old [28].

At national level, among the three editions of the World School Health Survey (EMSE, due to its acronym in Spanish), it can be observed an increase in the prevalence of overweight and obesity in adolescents between 13 and 15 years old, from 24.5% in 2007 to 28.6% in 2012, and to 33.1% in 2018; and from 4.4% in 2007 to 5.9% in 2012, and to 7.8% in 2018, respectively [28, 29]. In this particular case, the prevalence found were lower than in the EMSE 2012 y 2018, probably due to the prevalence of excess weight increases as age increases, in general.

At international level, in Perú during 2013–2014, a similar prevalence was observed (17.5% of overweight and 14.8% of obesity) in children between 5 and 9 years old in relation to the second period of this study [30]. Besides, in different age groups in China, an increase in overweight and obesity trends can be observed. Meanwhile, the prevalence of wasting decreased [11, 31]**; s**imilar to the results found in this study, where the excess weight increased and thinness decreased. These results confirm that in many countries, including Argentina, while the prevalence of excess weight is increasing, the prevalence of deficit malnutrition is decreasing [32].

This study also indicates sexual dimorphism (sex differences) in the prevalence of overweight and obesity, being higher mostly in girls, and in the magnitude of its increase, being significantly higher in boys in all areas and in both periods. This sex disparity in obesity can be explained by sociocultural, socio-economic, behavioral, ethnic and genetic factors [33]. However, in many western countries, where the prevalence of childhood obesity is higher in girls, it is partially attributed to the fact that boys have a significantly higher energy expenditure and resting metabolic rate than girls [33].

In the three EMSE and in the ENNyS 2018/9 carried out in Argentina, obesity were higher in males than in females [5, 29, 34]. A similar pattern was observed in a study with data of some years between 1987 and 2011, collected by the Spanish Ministry of Health and National Institute of Statistics: it showed that in boys and girls aged between 6 and 9, the prevalence of overweight rose, being higher in boys. Obesity remained steady with some fluctuations among boys, and it decreased among girls, being higher than in boys, though. In the youngest group, aged between 2 and 5, overweight and obesity remained similar for boys, with some fluctuations during this period. Similar patterns were seen among girls, among whom overweight remained relatively steady, whereas obesity decreased during this period [14]. Conversely, in the majority of Asian countries, particularly in China, there is a significantly higher trend of rise in obesity prevalence in boys compared to girls [33]. Brazil and Mexico are the Latin American countries where a higher prevalence of obesity in boys (5 to 11 years of age) than in girls was observed [35]. Regardless the country, national surveys, among others, show higher prevalence of obesity in boys. This can be attributed to the broader age intervals which include, in many cases, adolescence, stage in which growth pattern features are remarkable different between boys and girls. Besides, in general, these surveys have regional representativeness, blurring sociocultural, ethnic and economic peculiarities of the different cities.

In relation to the increase magnitude in the obesity prevalence in Argentina, in EMSE 2018 the increase magnitude of obesity prevalence in adolescents (13–15 years old) over 11 years (2007 to 2018) was around 77%, at the national level [29, 34]. Similar behavior was observed in the present study, where between the first and second period, the increase in the obesity prevalence is really alarming (115% for the total). In Perú, the obesity prevalence increased 92.2% in 7 years, from 2007 to 2014 [30]. On the other hand, in Shenyang (China) in 4 years it was on average 36.8% in boys and 41.6% in girls [11] and in another city of China, the increase in 9 years was around 100% [30].

Regarding to the magnitude of the increase in the prevalence of overweight, it was minor in relation to obesity. In agreement with this, a decrease on thinness and normalweight was observed. A similar patron was observed in Argentina, Perú and China [11, 29–31]. The differences between studies most likely reflect differing age groups, time periods, obesity definition, exclusion criteria, or subpopulation groups reflecting regional differences.

The stability of the prevalence of thinness associated with a greater increase in the prevalence of obesity observed in this study is consistent with research conducted at a national level that indicates that malnutrition due to excess weight almost tripled undernutrition [32]. Besides, with the hypothesis raised by Duran et al. (2006) which predicts in Latin American populations a positive secular trend in height with an increase in excess weight. In addition, this situation it may be also related to implementation of various food assistance programs focused on the undernutrition in conjunction with the nutritional transition evidenced in the country during the study period [36].

Several studies highlight the importance of assessing health together with the neighbourhoods in which people live. The conditions of these places reflect social contexts in which changes in individual behaviors such as physical inactivity, sedentary lifestyle and inadequate feeding, coupled with socioeconomic deprivation, poor housing conditions and lack of parks and recreation areas can lead to an increased risk of obesity [17, 37–40].

Historically in Argentina, social programs in general, and food programs in particular, are aimed to por families and specifically to children and pregnant women. However, such programs lack monitoring and assessment, which make them inefficient and unable to solve long term problems. To revert this situation, it is essential to reformulate its conception, content and extent in order to ensure the right to an adequate feeding with a real impact in the population’s health and nutrition [41].

As mentioned before, in San Salvador de Jujuy different levels of urban segregation are observed, dividing the urban space into three zones: some of these differences are lost within the zones [18, 19].

Besides, despite the spatial differences observed in nutritional status, the obesity prevalence is alarming and indicates that all areas are potentially obesogenic, being the center zone the most obesogenic. In this particular zone, the traditional houses of upper class families remain there, but there are also more unfavored areas such as villas, located on the banks of the river, where families with scarce resources live [19]. In addition, due to the large number of educational establishments that are concentrated in this zone, many schoolchildren attend to school there, regardless of where they reside. So, the population analyzed in this area is really diverse and this study could suggest just an association between the spatial distribution and the high prevalence of obesity in this area.

However, taking the UBN average of the 2010 census zones, it is observed that the highest UBN is that of the central zone (slightly higher than the others) and that coincides with the spatial segregation observed for obesity, where schoolchildren who attend school in this area have a greater chance of presenting it.

A similar study, conducted in Puerto Madryn (Argentina) in 2017, found that there were no significant variations of the UBN from 2001 to 2010 in the neighborhoods. In terms of nutritional status, regardless of the UBN, without significant and relevant variations in underweight, stunting and overweight, a significant increase in the prevalence of obesity was observed, similar to what was found in this study [40]. In conclusion, the magnitude of the influence of socioeconomic factors requires further study, by analyzing children’s residence area or the differences within the zones.

The results achieved in this work show that drawing public health attention to the health risks associated with the social structure and ecology of neighborhoods, innovative approaches to community level interventions may ensue [17].

The limitations of this work are associated with the use of secondary data sources where the measurement-record error cannot be controlled and the analysis is limited to the available variables, not including clinical information about the schoolchildren or complementary studies (like risk factors for obesity, overweight, and thinness).

On the other hand, the representativeness of the population and the reliability of the data taken by trained personnel are strengths of this study. Besides, it showed the epidemiologic characteristics of obesity, overweight, and malnutrition in schoolchildren in Jujuy, Argentina, which reflects the latest data available. This is beneficial to generate and adopt specific measures to prevent childhood obesity and malnutrition.

## Conclusion

The current study contributes with valuable information on the magnitude of the problematic of excess weight in the school population of Jujuy and suggests a possible correlation with the spatial distribution. Therefore, it supports the need for a new research agenda accompanied by a strengthened translation of the research into policy and practice and increased research capacity is needed in the region to confront this growing epidemic [3]. Taking this situation into account, it is essential to include economic and spatial distribution variables that allow a better understanding of the relationship between the environment where people live, study or work, and their health. The incorporation of the spatiality in the study of malnutrition enables the identification of critical areas which is key to allow epidemiologic surveillance and to lead policies that ensure food safety of all the population, specially most vulnerable ones and, in such way, reduce social inequalities related to the access to health, food education and adequate feeding.

## Data Availability

The datasets used and analyzed during the current study are not available on a free access website. Therefore, we cannot provide a link and access to the bases is closed. They were obtained by administrative steps of the research team before the competent authority of the School Health Program of Jujuy and they are available from the corresponding author on reasonable request. These are deposited in the digital file of the Departamento de Genética y Bioantropologia of the Instituto de Biología de la Altura (Jujuy, Argentina).

## Abbreviations

ENNyS: National Survey of Nutrition and Health (due to its acronym in Spanish)
UBN: Unsatisfied Basic Needs
BMI: Body mass index
EMSE: World School Health Survey (due to its acronym in Spanish)
RRR: Relative Risk Rate
CI: Confidence Intervals
